# Antimicrobial resistance in livestock and poor quality veterinary medicines

**DOI:** 10.2471/BLT.18.209585

**Published:** 2018-07-16

**Authors:** Katie Clifford, Darash Desai, Clarissa Prazeres da Costa, Hannelore Meyer, Katharina Klohe, Andrea S Winkler, Tanvir Rahman, Taohidul Islam, Muhammad H Zaman

**Affiliations:** a College of Engineering, Department of Biomedical Engineering, Boston University, 44 Cummington Mall, Boston, Massachusetts, United States of America.; b Center for Global Health, Institute of Medical Microbiology, Immunology and Hygiene, Technical University of Munich, Munich, Germany.; c Center for Global Health, Department of Neurology, Technical University of Munich, Munich, Germany.; d Department of Microbiology and Hygiene, Faculty of Veterinary Science, Bangladesh Agricultural University, Mymensingh 2202, Bangladesh.; e Department of Medicine, Bangladesh Agricultural University, Mymensingh, Bangladesh.

Every year, antimicrobial resistance causes the death of around 700 000 people, and this number is expected to rise to an estimated 10 million deaths annually by 2050.^1^ Antimicrobial resistance has the potential to affect almost all sustainable development goals (SDGs), particularly those targeting poverty, hunger, health and economic growth. Although the reduction and eradication of antimicrobial resistance is not included as an individual SDG, paragraph 26 of *Transforming our world: the 2030 agenda for sustainable development* states: “We will equally accelerate the pace of progress made in fighting malaria, [human immunodeficiency virus/acquired immunodeficiency syndrome], tuberculosis, hepatitis, Ebola and other communicable diseases and epidemics, including by addressing growing antimicrobial resistance and the problem of unattended diseases affecting developing countries.”^2^

There is concern about how antimicrobial resistance emergence in livestock will impact SDG 3, i.e. ensuring healthy lives and promoting wellbeing for all, at all ages.^3^ With meat production set to increase from 200 million tons to 470 million tons by 2050,^4^ it is likely that farmers will rely even more on antibiotics to prophylactically prevent disease in their livestock to meet this expected demand. The high proportion of poor-quality veterinary medicine for therapeutic use in livestock compounds the problem of antibiotic overuse, particularly in low- and middle-income countries. The annual market in Africa for substandard and non-registered veterinary medicine is estimated to be 400 million United States dollars,^5^ equal to that of the officially registered, quality-assured veterinary drug market.^6^

Numerous cases of antimicrobial resistance in humans have been traced to resistant microbes suspected of originating in livestock,^7^^,^^8^ which is particularly concerning as infected livestock can be asymptomatic.^8^ Transmission of resistant bacteria from livestock to humans can occur through the consumption of meat, direct contact with colonized animals or manure spread in the environment.^7^ The strongest correlation between interspecies pathogen transmission is observed in countries with policies to reduce agricultural antibiotic use. When the glycopeptide avoparcin was banned across the European Union in the late 1990s, the prevalence of vanco­mycin-resistant *enterococci* in biological samples from both poultry and humans decreased.^9^

While research on drug resistance, including the role of poor quality medicine, has increased in recent years, the scientific community has not incorporated the impact of poor-quality veterinary medicine into the overall picture of antimicrobial resistance. Poor-quality medicines that provide subtherapeutic doses of active pharmaceutical ingredient, whether due to inadequate amounts of pharmaceutical, ineffective release, presence of impurities or degradation of compounds, are believed to contribute to antimicrobial resistance by exposing microbes to a level of antibiotic that will not effectively kill the whole microbial population.^10^

Poor-quality veterinary medicine as a contributor to antimicrobial resistance has been mentioned anecdotally in the literature, but systematic studies are lacking. Given the widespread use of antibiotics in animal husbandry and the persistent problems of drug quality in low- and middle-income countries, this is a significant oversight that could lead to long-term challenges. Increased frequency of antibiotic use in livestock, particularly at subtherapeutic doses due to issues with administration and/or drug quality, can allow resistant microbes to flourish. While poor-quality veterinary medicine is presumed to impact antimicrobial resistance, there are no robust studies that can act as an evidence base for developing sound policies.

## Research and surveillance

In response to this knowledge gap, we recommend a multidisciplinary approach to investigate the role of poor-quality veterinary medicine on the emergence of antimicrobial resistance. The first step would be to identify areas where resistance, particularly to clinically important antibiotics such as quinolones, is found. Organizations such as the World Organization for Animal Health^11^ and the World Health Organization (WHO)^12^ maintain lists of antibiotics used in human and veterinary medicine, and rank their clinical importance for human health. However, an adequate active global surveillance and centralized database where antimicrobial resistance reports can be monitored does not exist. The WHO Global Antimicrobial Resistance Surveillance System provides a foundation for antimicrobial surveillance but does not currently collect data on microbes associated with livestock production.^13^ While 40 countries currently report to this surveillance system, several countries in the WHO Regions of Africa and the Americas do not. Expansion of surveillance to include livestock-related microbes and enrolment of additional low- and middle-income countries would help create a more complete picture of the role of animal production in antimicrobial resistance.

In areas where antimicrobial resistance is detected, surveys are needed to assess the quality of veterinary medicine and the procurement, prescribing and behavioural approaches to antibiotic supervision. Pharmaceutical quality is often assessed using high-performance liquid chromatography on field samples, an expensive, laboratory-based test that requires well-trained staff and a long waiting period for results.To sufficiently survey veterinary medicine quality in low- and middle-income countries, portable technologies that can provide quantitative information about the active pharmaceutical ingredient in an efficient and affordable manner are needed.

## Quality assurance

### Pre-market identification

Veterinary medicine follows many channels and depends on many players in the supply chain before reaching the consumer. There are many points where quality can be compromised and often there are no adequate systems to monitor distribution.^14^ For both domestic manufacturing and international importation of veterinary medicine, governments could hold manufacturers to high standards and be prepared to levy fines or reject shipments should the medicine lot fail quality checks. While this action would likely require coordination with and funding support for customs, stopping poor-quality veterinary medicine products at entry is less expensive and less challenging than product recall. New technologies to assess medicine quality in the field at a reasonable cost would reduce the barriers facing authorities tasked with testing at domestic manufacturing plants and ports of entry, making rapid on-site testing a more attractive option.

The World Organization for Animal Health offers guidance to support countries working towards implementing quality assurance systems for veterinary medicine. The International Cooperation on Harmonization of Technical Requirements for Registration of Veterinary Medicinal Products programme develops global standards for veterinary medicine and helps countries establish drug registration protocols.^15^ The World Organization for Animal Health National Focal Point for Veterinary Products has established a network of global veterinary product experts, with each member nation represented by a local authority on veterinary health and medicine.^16^

### Post-market identification

Organizations concerned with antimicrobial resistance should work to raise awareness among government stakeholders in low- and middle-income countries about the issues surrounding poor-quality veterinary medicine, encouraging these countries to prioritize secure supply chains for veterinary medicine. Innovative solutions for medicine supply chain management are at the forefront of many global health initiatives; this is a prime opportunity to ensure these improvements are also incorporated for veterinary medicine supply chains.

Portable technologies that can rapidly assess medicine quality would allow regulatory agents to test and take immediate action at point of testing, therefore preventing poor-quality veterinary medicine from entering the market. To have a sustainable impact, post-market testing must be paired with strong regulations, fines and prosecution for repeat offenders.

### Reporting and data dissemination

With portable, affordable drug testing technologies allowing for rapid assessment of medicine quality, data on veterinary medicine quality can be collected throughout the supply chain. To incorporate this data into a usable format, a centralized, accessible and transparent government system for veterinary medicine quality reporting should be created. This would provide the information that policymakers and government officials need to make impactful regulations and decisions. Information collected on medicine quality must also be effectively communicated to all farmers purchasing veterinary medicine. However, for farmers to make informed purchasing decisions, they must have access to quality veterinary medicine, which requires secure supply chains and financial accessibility.

## Incentivizing farmers

Disincentivizing the production and sale of poor-quality veterinary medicine is an essential element in antimicrobial resistance reduction. However, this action needs to be paired with policies that encourage farmers to purchase quality veterinary medicine, because the poor-quality veterinary medicine problem is linked to the intense demand for inexpensive livestock medication.

A deeper understanding of antimicrobial consumption trends in livestock is needed, such as sourcing of medication and the scale of treatment using antibiotics. The World Organization for Animal Health and the European Union, (via the European Surveillance of Veterinary Antimicrobial Consumption) are currently collecting data on the use of antimicrobials in animals for consumption.^17^^,^^18^ While this data will reveal the magnitude of the problem, information on the social and behavioural aspects of veterinary practice is needed to craft effective policy to disincentivize the purchase of poor-quality veterinary medicine. Surveys among farmers on the reasons for purchasing unregulated medicine and on their understanding of the short- and long-term consequences, would inform awareness campaigns and could help developing better policies.

Disincentives can also take the shape of embargos set by importing nations on meat products exported from countries where there is a high prevalence of poor-quality veterinary medicine used in livestock. Again, these disincentives are based on the decision-making power of rural farmers to purchase quality medicines, which requires strong national supply chains for veterinary medicine and financial and geographical accessibility.

## Conclusion

Investigating the role of poor quality veterinary medicines as a driver of antimicrobial resistance will provide stakeholders with a deeper understanding of antimicrobial resistance emergence and of how interrelated factors foster an environment from which resistance arises. Producing an impact will require governments, global organizations, medicine suppliers, farmers and meat consumers to act on that newfound understanding. Each of these stakeholders will have to make changes, within their respective roles, to ensure appropriate use of quality veterinary medicine to tackle antimicrobial resistance and ensure good health and wellbeing for all.

## References

[1] Tackling drug-resistant infections globally: final report and recommendations. London: The Review on Antimicrobial Resistance; 2016. Available from: https://amr-review.org/sites/default/files/160525_Final%20paper_with%20cover.pdf

[2] Resolution A/RES/70/1. Transforming our world: the 2030 agenda for sustainable development. In: Seventieth United Nations General Assembly, New York, 25 September 2015. New York: United Nations; 2015. Available from: http://www.un.org/ga/search/view_doc.asp?symbol=A/RES/70/1&Lang=E [cited 2018 May 24].

[3] Antimicrobial resistance [internet]. Geneva: World Health Organization; 2018. Available from: http://www.who.int/news-room/fact-sheets/detail/antimicrobial-resistance [cited 2018 May 24].

[4] 2050: A third more mouths to feed. Rome: Food and Agriculture Organization of the United Nations; 2009. Available from: http://www.fao.org/news/story/en/item/35571/icode/ [cited 2018 June 21].

[5] Kingsley P. How fake animal medicines threaten African livestock. Geneva: World Economic Forum; 2015 Available from: https://www.weforum.org/agenda/2015/02/how-fake-animal-medicines-threaten-african-livestock/[cited 2018 Jun 12].

[6] Alliance to combat black market in counterfeit veterinary drugs. Rome: Food and Agriculture Organization of the United Nations; 2012. Available from: http://www.fao.org/news/story/en/item/123165/icode/ [cited 2018 May 24].

[7] Paphitou NI. Antimicrobial resistance: action to combat the rising microbial challenges. Int J Antimicrob Agents. 2013 6;42 Suppl:S25–8. 10.1016/j.ijantimicag.2013.04.007 23684003

[8] Mamun M, Hassan J, Nazir K, Islam A, Zesmin K, Rahman B, et al. Prevalence and molecular detection of quinolone-resistant E. coli in rectal swab of apparently healthy cattle in Bangladesh. Int J Trop Dis Health. 2017;24(2):1–7. 10.9734/IJTDH/2017/34404

[9] Klare I, Badstübner D, Konstabel C, Böhme G, Claus H, Witte W. Decreased incidence of VanA-type vancomycin-resistant enterococci isolated from poultry meat and from fecal samples of humans in the community after discontinuation of avoparcin usage in animal husbandry. Microb Drug Resist. 1999 Spring;5(1):45–52. 10.1089/mdr.1999.5.45 10332721

[10] Zaman M. Bitter pills: the global war on counterfeit drugs. Oxford: Oxford University Press; 2018.

[11] Guidelines and resolution on antimicrobial resistance and the use of antimicrobial agents. Paris: World Organization for Animal Health; 2015 Available from: https://web.oie.int/delegateweb/eng/ebook/AF-book-AMR-ANG_FULL.pdf?WAHISPHPSESSID=03152ead00d06990fa9066b7b71fcabc

[12] Critically important antimicrobials for human medicine. Geneva: World Health Organization; 2017. Available from: http://www.who.int/foodsafety/areas_work/antimicrobial-resistance/cia/en/ [cited 2018 May 23].

[13] Tornimbene B, Eremin S, Escher M, Griskeviciene J, Manglani S, Pessoa-Silva CL. WHO Global Antimicrobial Resistance Surveillance System early implementation, 2016–17. Lancet Infect Dis. 2018 3;18(3):241–2. 10.1016/S1473-3099(18)30060-4 29396007

[14] Sommanustweechai A, Chanvatik S, Sermsinsiri V, Sivilaikul S, Patcharanarumol W, Yeung S, et al. Antibiotic distribution channels in Thailand: results of key-informant interviews, reviews of drug regulations and database searches. Bull World Health Organ. 2018 2 1;96(2):101–9. 10.2471/BLT.17.199679 29403113 PMC5791780

[15] International Cooperation on Harmonisation of Technical Requirements for Registration of Veterinary medicinal Products 9VICH. London: European Agency for the Evaluation of Medicinal Products; 2018. Available from: http://www.vichsec.org/ [cited 2018 May 22].

[16] National Focal Points for Veterinary Products. Paris: World Organization for Animal Health; 2013. Available from: http://www.rr-asia.oie.int/about-us/focal-points/veterinary-products/ [cited 2018 May 22].

[17] OIE annual report on antimicrobial agents intended for use in animals: better understanding of the global situation, second report. Paris: World Organization for Animal Health; 2017. Available from: http://www.oie.int/fileadmin/Home/eng/Our_scientific_expertise/docs/pdf/AMR/Annual_Report_AMR_2.pdf [cited 2018 May 24].

[18] European Surveillance of Veterinary Antimicrobial Consumption (ESVAC). London: European Medicines Agency, 2010. Available from: http://www.ema.europa.eu/ema/index.jsp?curl=pages/regulation/document_listing/document_listing_000302.jsp&mid=WC0b01ac0580153a00 [cited 2018, May 22].

